# Supplementary material for: Measuring health-related quality of life of care home residents, comparison of self-report with staff proxy responses for EQ-5D-5L and HowRu: protocol for assessing proxy reliability in care home outcome testing
Source: BMJ Open. 2018 Aug 17;8(8):e022127. doi: 10.1136/bmjopen-2018-022127 (PMC6104798; doi:10.1136/bmjopen-2018-022127)
Supplement: Author's manuscript [file bmjopen-2018-022127.draft_revisions.pdf]

BMJ Open

BMJ Open is committed to open peer review. As part of this commitment we make the peer review history of every article we publish publicly available.

When an article is published we post the peer reviewers' comments and the authors' responses online. We also post the versions of the paper that were used during peer review. These are the versions that the peer review comments apply to.

The versions of the paper that follow are the versions that were submitted during the peer review process. They are not the versions of record or the final published versions. They should not be cited or distributed as the published version of this manuscript.

BMJ Open is an open access journal and the full, final, typeset and author-corrected version of record of the manuscript is available on our site with no access controls, subscription charges or pay-per-view fees (http://bmjopen.bmj.com).

If you have any questions on BMJ Open's open peer review process please email info.bmjopen@bmj.com

BMJ Open

Measuring health related quality of life of care home residents, comparison of self-report with staff proxy responses for EQ-5D-5L and HowRu: Protocol for the APRICOT (Assessing Proxy Reliability In Care home Outcome Testing) Study

Journal:	*BMJ Open*
Manuscript ID	bmjopen-2018-022127
Article Type:	Protocol
Date Submitted by the Author:	02-Feb-2018

Complete List of Authors:	Usman, Adeela; University of Nottingham, Division of Medical Sciences and Graduate Entry Medicine<br>Lewis, Sarah; University of Nottingham, Division of Epidemiology and Public Health<br>Hinsliff-Smith, Kathryn; University of Nottingham, Division of Medical Sciences and Graduate Entry Medicine<br>Long, Annabelle; University of Nottingham, Division of Medical Sciences and Graduate Entry Medicine<br>Housley, Gemma; East Midlands Academic Health Sciences Network<br>Jordan, Jake; University of Surrey, School of Economics<br>Gage, Heather; University of Surrey, School of Economics<br>Dening, Tom; University of Nottingham,<br>Gladman, John; University of Nottingham, Division of Rehabilitation and Ageing<br>Gordon, Adam; University of Nottingham, Division of Medical Sciences and Graduate Entry Medicine; City, University of London, School of Health Sciences

Keywords:	Quality of Life, Homes for the Aged, Frailty

SCHOLARONE™
Manuscripts

Measuring health related quality of life of care home residents, comparison of self-report with staff proxy responses for EQ-5D-5L and HowRu: Protocol for the APRICOT (Assessing Proxy Reliability In Care home Outcome Testing) Study

Adeela Usman [1]
Sarah Lewis [2]
Kathryn Hinsliff-Smith [1]
Annabelle Long [1]
Gemma Housley [3]
Jake Jordan [4]
Heather Gage [4]
Tom Dening [5]
John R F Gladman [6, 7]
Adam L Gordon [1, 8]

1. Division of Medical Sciences and Graduate Entry Medicine, School of Medicine, University of Nottingham, UK.
2. Division of Epidemiology and Public Health, School of Medicine, University of Nottingham, UK.
3. East Midlands East Midlands Academic Health Science Network, Nottingham, UK.
4. School of Economics, University of Surrey, UK.
5. Institute of Mental Health, University of Nottingham, UK.
6. Division of Rehabilitation and Ageing, University of Nottingham, UK
7. East Midlands Collaboration for Leadership in Applied Health Research and Care, UK
8. School of Health Sciences, City, University of London, UK.

Corresponding Author: Adam Gordon, Division of Medical Sciences and Graduate Entry Medicine, University of Nottingham, Room 4113, Derby Medical School, Derby. DE22 3NE.
Email: adam.gordon@nottingham.ac.uk; Tel: 01332 724668; Fax: 01332 724697

Word Count: 2793 words

For peer review only

Abstract

Introduction

Research into interventions to improve health and wellbeing for older people living in care homes is increasingly common. Health-Related Quality of Life (HRQoL) is frequently used as an outcome measure but collecting both self-reported and proxy HRQoL measures is challenging in this setting. This study will investigate the reliability of UK care home staff as proxy respondents for the EQ-5D-5L and HowRu measures.

Methods and Analysis

This is a prospective cohort study of a sub-population of care home residents recruited to the larger **P**roactive H**e**althc**a**re for Older People in **C**are **H**omes (PEACH) study. It will recruit residents ≥ 60 years across 24 care homes with mental capacity to consent to participation and not receiving short stay or respite care. The sample size is 158 participants. Resident and care home staff proxy EQ-5D-5L and HowRu responses will be collected monthly for three months. Weighted kappa statistics and intraclass correlation adjusted for clustering at the care home level will be used to measure agreement between resident and proxy responses. The extent to which staff variables (gender, age group, length of time caring, role, how well they know the resident, length of time working in care homes and in specialist gerontological practice) influence the level of agreement between self-reported and proxy responses will be considered using a multilevel mixed-effect regression model.

Ethics and Dissemination

This study is part of preparatory work for the larger PEACH study. The UK Health Research Authority and the University of Nottingham Research Ethics Committee determined this to be a service development and evaluation project. The PEACH study protocol has been reviewed as part of good governance by the Nottinghamshire Healthcare Foundation Trust. We will publish this study in a peer-reviewed journal with international readership and disseminate it through relevant national stakeholder networks and specialist societies.

For peer review only

For peer review only

Introduction

Long term care facilities in the UK are called care homes, and are classified as either care homes with or without nursing based upon the availability of registered nurses on-site (1). The types of residents cared for in both classifications of facility are similar and all UK care homes are included in the international consensus definition of a nursing home (2). Around 425,000 people live in care homes in the UK (3) with most residents requiring care due to disability from long-term conditions. The majority of residents are aged over 85, 75-80% of residents live with dementia (4), and over half of residents die within 12 months of admission to care home (5).

Improving the quality of care for older people in long-term care has become a focus of attention for health and social care providers, both within the UK and internationally (2), and an increasing number of evaluative research studies are being published that test the effectiveness and cost-effectiveness of interventions in this setting. Residents' quality of life (QoL) is frequently used as an outcome measure in these studies both to maintain a patient-centred focus and to facilitate health economic evaluation. The prevalent frailty and cognitive impairment in the care home population, however, means collecting self-reported QoL measures from residents is challenging. As a response to this, proxy responses to QoL items have sometimes been used. For these a consultee, drawn from one of care home staff, or a relative or friend, answers questions on the resident's behalf. Using proxy respondents can be unreliable in care home settings. There may be lack of continuity of care home staff contact with individual residents due to shift working and staff turnover, and family and friends may not be well placed to judge QOL domains if they visit residents for only short periods (6).

The EurQoL questionnaire is a widely-used preference-based health-related quality of life (HRQoL) measure suitable for use in economic evaluations. The EQ-5D-5L version measures HRQoL across five domains (mobility, self-care, usual activities, pain, anxiety/depression) with the scale for each domain ranging from level 1 (no problems) to level 5 (extreme problems). The responses from the five domains are converted to QoL index scores (utilities) generated from a given country's general population (7). These index scores can be used to calculate quality adjusted life years (QALYs), which are a measure of the person's state of health – one QALY equates to one year in perfect health. QALYs are calculated using the area under the curve (8) defined by utility scores at the different assessment points over the study period. The cost per QALY gained from an intervention when compared to usual care is the chosen cost-utility measure for determining eligibility for public support of the intervention through the UK National Health Service (9).

The scale for the first version of EQ-5D had only three levels (EQ-5D-3L). EQ-5D-3L has been shown to have good construct validity for self-report (10), and has been used to measure QoL of older people living in their own homes and in care homes (11). The 5L version was developed subsequently to improve responsiveness. There is also an EQ-5D visual analogue scale (VAS) for assessing the overall health status at a point in time, ranging from 0 (worst imaginable) to 100 (best imaginable), but this has been shown to have poor reliability (12).

It is recognised that the EQ-5D, in all its forms, is limited by consequence of being a generic measure that fails to take account of the difference in what constitutes "quality of life" in a long-term care setting. It doesn't take account of shifts in emphasis about what constitutes wellbeing as residents enter long-term care, which means that social care related quality of life (SCRQoL) measures such as the Adult Social Care Outcomes Toolkit (ASCOT) may be preferable in this setting. A further

For peer review only

critique has been that it fails to separate capability (what a resident is able to do) from preference (what a resident chooses to do under the circumstances), with the result that some authors have championed capability-based outcome measures, such as the ICEpop Capability Measure for Older People (ICECAP-O), in care homes (13-15). Best practice suggests that, if EQ-5D is used in this setting, it is used in combination with more specific instruments.

The R-outcomes – howRu and howRwe – have been specifically designed for use in long term care settings in order to address quality of life and quality of care in a straightforward way that is practical with older people. HowRu is a Patient Recorded Outcome Measure (PROM) that records four variables (pain or discomfort, feeling low or worried, limitation in activities, and dependency on others) related to quality of life at a fixed point in time ("How are you doing today?") on a four-point scale (none, slight, quite a lot, extreme) (16). The HowRU score is calculated by summing up the values for each domain to give a value on a 13-point scale ranging from 0 (worst) to 12 (best). PROMS, like the R-outcomes, have been specifically designed with older adults in mind, and may have a cogency and immediacy that improves upon some of the measurement uncertainty introduced by the relative abstraction of the questions included in highly validated general population indices such as EQ-5D-5L.

In a comparison with EQ-5D in patients attending a cardiovascular outpatient clinic, HowRU was reported to have better readability, higher completion rate, wider range of states used and smaller ceiling effect (17). No national tariffs exist to enable calculation of HowRU indices that would facilitate its use as a preference-based measure in cost-utility analysis. Understanding how and whether R outcomes correlate with EQ-5D scores in the care home setting is, however, helpful when considering additional information that can help triangulate our understanding of how interventions affect quality of life in this context. Knowing that HowRu correlates with EQ-5D may provide further justification for using it in clinical settings where broad judgements about impact on quality of life have to be made without the need for detailed cost-utility analysis.

Proxies have been used to capture EQ-5D-3L responses from people with dementia, although poor agreement between patient and proxy ratings has raised concerns (7,8) and differences between professional and family carer ratings have led to questions about the appropriate choice of proxy (9). In a comparison of clinicians and family carers as proxies, clinician responses had better construct validity in the more observable domains of mobility and self-care, and family carer responses had better construct validity in the less observable domains of usual activities and anxiety/depression (9). There is limited evidence, however, comparing self-reported and proxy responses to the EQ-5D-5L in care home populations (10). There is, in particular, a paucity of data as to how it performs in UK care home populations. This is important because institutional care is structured differently between nations, with differing professional carer competencies, patterns of working and job roles. This means that carers in different countries will have differential exposure to residents and different competencies in terms of their ability to interpret residents' experiences, and a tool that works for professional proxy response in the US may not, therefore, work as well in the UK.

The HowRu, as a recently developed PROM, is yet to be fully evaluated for older people living in care homes (18). It is not known whether proxy responses in this setting may be useful in completing HowRU and there are no data on how HowRu correlates with EQ-5D in the care home populations.

This study seeks to fill these evidence gaps.

Aim

The Assessing Proxy Reliability In Care home Outcome Testing (APRICOT) study is a preparatory piece of work for the Proactive Healthcare for Older People Living in Care Homes (PEACH) study. It aims to examine the level of agreement between the responses to EQ-5D-5L and HowRu by care home staff and residents, and between EQ-5D-5L and HowRu as quality of life measures. Findings will assist in the interpretation of quality of life data gathered for the larger PEACH study.

Objectives

To determine the level of agreement between:

- Resident EQ-5D-5L and staff-proxy EQ-5D-5L responses
- Resident HowRu and staff-proxy HowRu responses
- Resident EQ-5D-5L and HowRu responses
- Proxy EQ-5D-5L and HowRu responses

Methods

Setting

24 care homes in the East Midlands area of England.

Brief description of the Proactive Healthcare in Older Peoples' Care Homes (PEACH) study

The PEACH intervention involves using Quality Improvement Collaboratives as a mechanism to encourage implementation of Comprehensive Geriatric Assessment (CGA) as a unifying framework for assessment and delivery of healthcare in UK care homes. CGA is widely recognised as a gold-standard way to deliver care for older people with frailty (19). The aim of PEACH is to improve quality of care and quality of life for care home residents. Outcome data quantifying healthcare resource use and resident level quality of life will be collected on a monthly basis to assess the impact of the intervention.

Two instruments are being used in PEACH to assess residents' quality of life, the EQ-5D-5L and HowRu. The rationale is that these reflect measurable differences in the patient experience that may translate, with some interpretation, into an understanding of how CGA influences quality of care and general wellbeing. APRICOT has been designed as a preparatory sub-study within PEACH to better enable interpretation of proxy EQ-5D-5L and HowRU responses collected as part of outcome measurement.

Participants

Care home recruitment for PEACH took place between October 2016 and January 2017, with individual resident recruitment from January 2017. A prospective cohort of a sub-population of residents will be included in the comparison of proxy and self-report measurement of EQ-5D-5L and HowRu in APRICOT. Residents without mental capacity to provide consent will be excluded from the study. This is because we want to compare proxies against a gold standard which is represented by self-response in residents who have capacity, where cognitive impairment will not confound their responses regarding QoL. When managers are unsure, researchers will make judgements based on the framework for mental capacity outlined in the 2005 Mental Capacity Act for England and Wales (20).

This study will be conducted in parallel to the main PEACH study. In addition to the routine collection of EQ-5D-5L and HowRu from residents recruited to PEACH, proxy responses to EQ-5D-5L and HowRu will be gathered from staff that provide regular

For peer review only - http://bmjopen.bmj.com/site/about/guidelines.xhtml

personal care in their day-to-day role. Care home staff acting as proxies may include care workers, registered nurses, and care home managers. Staff employed in a supportive role such as activity coordinators will be excluded since their orientation to supporting residents is more variable.

Data collection

Data from proxies will be collected on three consecutive months. Due to the high staff turnover amongst care home staff, and to enable the influence of carer characteristics on the level of agreement to be estimated, data on the carer will be gathered at each assessment. Repeated measures are required for the final analysis in the PEACH study for calculating costs per QALY gained (comparing the intervention with usual care condition) and understanding how the reliability of proxies changes over subsequent measurements will therefore be important.

The EQ-5D standard operating procedure will be adopted to guide the proxy data collection procedure. Staff proxies will be asked to consider the proxy-resident's perspective when completing the questionnaire using the following statement: "*The following question requires you to respond in accordance to how you think the resident would rate their own general health state at the moment. By placing a tick in one box in each group, please indicate which statement best describes how you think the resident would assess their health state today"*(13). Both self-reported and proxy EQ-5D will be completed on the same day to minimize any variations in responses.

The EQ-5D VAS will be used in the study for completeness because it is part of the EQ-5D-5L instrument, even though there are concerns about its reliability (12).

Sample size calculation

When the sample size is 160, a two-sided 95.0% confidence interval for the intraclass kappa will extend 0.153 from the observed value of kappa, assuming that the true value of the intra-class kappa is 0.145 and the proportion of successes is 0.500.

Primary analysis

An overall agreement between the self-reported and staff proxy responses on the domain levels of the EQ-5D-5L and HowRu will be computed. Weighted kappa statistic and Intra-class correlation (ICC) will be used to calculate the level of agreement for categorical and continuous outcomes respectively. All reliability indices will be calculated at the domain levels and overall index scores/QALYs for the EQ-5D-5L.

Weighted kappa helps to distinguish between small and large difference in agreement ratings assigned to the different levels of each domain but equal importance is given to disagreement (21, 22). The weighting for kappa will be done using linear weight - this assigns the same importance to the difference between any two categories within the response scale (23). The confidence interval for the weighted kappa will be calculated by bootstrapping in Stata 15 (Statcorp, LLC, 2015) with 1000 replications. This will be done for each time point.

The kappa statistic ranges from -1 to 1, and the strength of the agreement will be interpreted with regards to published guidelines (24) with agreement being:

- Poor, if kappa ≤ 0.00
- Slight, if kappa = 0.00 to 0.20
- Fair, if kappa = 0.21 to 0.40
- Moderate, if kappa = 0.41 to 0.60

- Substantial, if kappa = 0.61 to 0.80
- Almost perfect, if kappa ≥ 0.80

Unadjusted ICC will be calculated using two-way mixed effect model to examine the level of agreement between the self-reported and proxy responses for the EQ-5D-VAS, EQ-5D index scores and total QALYs. The Bland-Altman graph (plotting the mean difference between the EQ-5D-5L-S (self-report) and EQ-5D-5L-P (proxy) against the mean of the two measures) will be constructed to supplement the ICC if data is found to be normally distributed. Data will be transformed to an approximately normal distribution if this is not initially the case.

Analysis will be done at each time point for Kappa and ICC. However, a single ICC value for QALYs will be calculated for individuals with data on all three consecutive months as this will be used in practice in the PEACH study, where analysis will be done on consecutive measures made over time.

To allow for comparability of the level of agreement at the domain and index score level; the same benchmarks used for kappa will be used for the ICC.

Clustering

Clustering will be adjusted for because the calculation of kappa and ICC assumes independence of observations. In our study, clustering could occur at three levels at each time point. Firstly, at the care home level, where residents within the same care home have similar characteristics and are different from those in other care homes. Secondly, at the individual level, where responses are clustered within each resident and lastly, at staff level, where staff members within a care home responds on behalf of multiple residents.

The ICC value will be calculated allowing for clustering using a nested two-way mixed effect model calculated by fitting a 2 level random effect model with a random effect for care home and individuals.

A cluster adjusted kappa will be calculated using a variance formula (13). This will include calculating kappa and its variance for each care home, then estimating the within cluster variance σ_{ω}^{2}using the average of the individual variances, and between cluster variance σ_{b}^{2}using the variance of the individual kappa. Using these estimates the clustered Kappa and its variance will be calculated using the formulas below:

$$\omega_{i} = \frac{n_i}{(1+(n_i-1)\rho_k)}$$

Where

n_i = Size of cluster i

ρ = Intra-cluster correlation coefficient for Kappa

$$\rho_k = \frac{\sigma_b^2}{(\sigma_\omega^2 + \sigma_b^2)}$$

Variance of the cluster adjusted kappa will be obtained using the equation below (24):

$$\sigma^2_{clustered\ k} = \frac{\sum_{i=1}^{k} \omega_i^2 \left(\sigma_b^2 + \frac{\sigma_\omega^2}{n_i}\right)}{\left(\sum_{i=1}^{k} \omega_i\right)^2}$$

For peer review only

The clustered kappa and its variance will then be divided by the square root of the number of individuals to get the standard error. The 95% confidence interval will be calculated using this standard error.
Cluster adjusted kappa (EQ-5D-5L and HowRu domain levels) and ICC (EQ-VAS and EQ-5D index scores) will be reported for each time point. However, QALYs will be presented over time, three months, similar to how it will be calculated in the PEACH study.

Secondary analyses
The effect of age, sex and length of stay in care home (for residents), length time working in care of older people/care homes and role/rank (for staff) at baseline on the difference between staff and proxy EQ-5D-5L-S scores will be analysed using a multilevel mixed effect regression model.

To investigate the reliability of using HowRu as a quality of life measure in the care home population compared with EQ-5D-5L, we will assess agreement between these indices using weighted kappa statistics. This will involve testing the level of agreement between domains with similar construct on both scales (25) as shown in table 1.

Table 1 - Planned pair-wise alignment of HowRU and EQ-5D-5L domains for agreement analysis using kappa

HowRu domains	EQ-5D-5L domains
Pain or discomfort	Pain/discomfort
Feeling low or worried	Anxiety/depression
Limited in what you can do	Mobility<br>Self-care<br>Usual activities
Dependent on others	Mobility<br>Self-care<br>Usual activities

Strengths and Limitations of this study

- To our knowledge, this is the first study to investigate the reliability of staff proxy using the EQ-5D-5L and HowRu, and the reliability of HowRu as a quality of life measure in a UK care home setting.
- The scales will be administered to residents and staff on the same day, thus reducing any variations that will affect the level of agreement.
- Analysing the characteristics of staff respondents will help generate insights into how proxies might better be selected or prepared to maximise the validity of proxy responses.
- The use of multiple time points will help increase the sample size and account for the influence of staff turnover on the quality of proxy-response.
- Working with residents with mental capacity will optimise their responses as a gold standard against which proxies can be compared. The way in which staff interpret quality of life in this more communicative and able group may, however, be different to the way in which the interpret quality of life in advanced dementia.

Ethics and dissemination

For peer review only

This study is part of preparatory work for the larger PEACH study. PEACH was reviewed by both the UK Health Research Authority and the University of Nottingham Research Ethics Committee and determined by both to be a service development and evaluation project. The PEACH study protocol has been reviewed as part of good governance by the Nottinghamshire Healthcare Foundation Trust. We aim to publish this study in a peer-reviewed journal with international readership and disseminate it further using relevant national stakeholder networks and specialist societies.

Funding
This work has been conducted as part of the Proactive Healthcare of Older People in Care Homes (PEACH) study, supported by the Dunhill Medical Trust, award number FOP1/0115.

Contributors
All authors meet the ICJME criteria for authorship. AU, SL, KHS, GH, JJ, HG, TD, JRFG and ALG conceived the study at a PEACH study management meeting and specified the aims and objectives. AU, SL and ALG produced the initial draft of the protocol, with KHS, GH, JJ, HG, TD and JRFG contributing to subsequent redrafts. AU and SL led on aspects of statistical design. JJ and HG provided specialist health economics input. AU, SL, KHS, GH, JJ, HG, TD, JRFG and ALG all reviewed the final manuscript and approved it prior to submission.

Competing interests
None declared.

Acknowledgements
The authors would like to acknowledge the broader PEACH study team: Mr Zimran Alam, Ms Anita Astle, Professor Tony Avery, Dr Jaydip Banerjee, Professor Clive Bowman, Dr Neil H Chadborn, Mr Michael Crossley, Dr Reena Devi, Professor Claire Goodman, Professor Pip Logan, Professor Finbarr Martin, Professor Julienne Meyer, Dr Dominick Shaw, Professor David Stott and Dr Maria Zubair.

References

1. Aspden T, Bradshaw SA, Playford ED, Riazi A. Quality-of-life measures for use within care homes: a systematic review of their measurement properties. Age and Ageing. 2014; 43(5):596-603.
2. Sanford AM, Orrell M, Tolson D, Abbatecola AM, Arai H, Bauer JM, et al. An International Definition for “Nursing Home”. Journal of the American Medical Directors Association. 2015;16(3):181-184.
3. Laing & Buisson. Care of the Elderly People Market Survey. London: Laing and Buisson 2010; 2009.
4. Gordon AL, Franklin M, Bradshaw L, Logan P, Elliott R, Gladman JRF. Health status of UK care home residents: a cohort study. Age and Ageing. 2014;43(1):97-103.
5. Kinley J, Hockley J, Stone L, Dewey M, Hansford P, Stewart R, et al. The provision of care for residents dying in UK nursing care homes. Age and Ageing. 2014;43(3):375-379.
6. Kane RL, Kane RA, Bershadsky B, Degenholtz H, Kling K, Totten A, et al. Proxy sources for information on nursing home residents' quality of life. The journals of

For peer review only

gerontology Series B, Psychological sciences and social sciences. 2005;60(6):S318-s325.
7. Dolan P, Gudex C, Kind P, Williams A. A social tariff for EuroQol: Results from a UK general population survey. The University of York Centre for Health Economics; 1995. [Accessed 7 December 2017]. Discussion Paper 138. Available from: http://www.york.ac.uk/che/pdf/DP138.pdf.
8. Matthews JN, Altman DG, Campbell MJ, Royston P. Analysis of serial measurements in medical research. BMJ. 1990;300(6719):230-235.
9. McCabe C, Claxton K, Culyer AJ. The NICE Cost-EffectivenessThreshold. Pharmacoeconomics. 2008;26(9):733-744.
10. McDowell I Newell C. Measuring health : a guide to rating scales and questionnaires. New York: Oxford University Press; 1996.
11. Borowiak E, Kostka T. Predictors of quality of life in older people living at home and in institutions. Aging Clinical and Experimental Research. 2004;16(3):212-220.
12. Hounsome N, Orrell M, Edwards RT. EQ-5D as a Quality of Life Measure in People with Dementia and Their Carers: Evidence and Key Issues. Value in Health. 2011;14(2):390-399.
13. Devine A, Taylor SJC, Spencer A, Diaz-Ordaz K, Eldridge S, Underwood M. The agreement between proxy and self-completed EQ-5D for care home residents was better for index scores than individual domains(). Journal of Clinical Epidemiology. 2014;67(9):1035-1043.
14. Bulamu NB, Kaambwa B, Ratcliffe J. A systematic review of instruments for measuring outcomes in economic evaluation within aged care. Health and Quality of Life Outcomes. 2015;13(1).
15. van Leeuwen KM, Bosmans JE, Jansen AP, Hoogendijk EO, van Tulder MW, van der Horst HE, et al. Comparing Measurement Properties of the EQ-5D-3L, ICECAP-O, and ASCOT in Frail Older Adults. Value in health : the Journal of the International Society for Pharmacoeconomics and Outcomes Research. 2015;18(1):35-43.
16. Benson T, Williams DH, Potts HWW. Performance of EQ-5D, howRu and Oxford hip & knee scores in assessing the outcome of hip and knee replacements. BMC Health Services Research. 2016;16(1).
17. Benson T, Potts HWW, Whatling JM, Patterson D. Comparison of howRU and EQ-5D measures of health-related quality of life in an outpatient clinic. Inform Prim Care. 2013;21(1):12-17.
18. Hendriks SH, Rutgers J, van Dijk PR, Groenier KH, Bilo HJG, Kleefstra N, et al. Validation of the howRu and howRwe questionnaires at the individual patient level. BMC Health Services Research. 2015;15(1).
19. Welsh TJ, Gordon AL, Gladman JR. Comprehensive geriatric assessment - a guide for the non-specialist. *Int J Clin Pract*. 2014. 68(3): 290–293
20. Dobson C. Conducting research with people not having the capacity to consent to their participation. A practical guide for researchers [Internet]. Leicester, UK: The British Psychological Society; 2017 [cited 1 December 2017]. Available from: https://pdfs.semanticscholar.org/f97c/5a3d8605e24eaaf965b64e42c690d42a6182.pdf
21. Vanbelle S, Albert A. A note on the linearly weighted kappa coefficient for ordinal scales. Statistical Methodology. 2009; 6(2):157-163.
22. Brenner H, Kliebsch U. Dependence of Weighted Kappa Coefficients on the Number of Categories. Epidemiology. 1996;7(2):199-202.
23. Cicchetti DV, Allison T. A New Procedure for Assessing Reliability of Scoring EEG Sleep Recordings. American Journal of EEG Technology. 1971;11(3):101-110.

For peer review only

24. Landis JR, Koch GG. The measurement of observer agreement for categorical data. Biometrics. 1977;33(1):159.
25. Kerry SM, Bland JM. Unequal cluster sizes for trials in English and Welsh general practice: implications for sample size calculations. Statistics in Medicine. 2001;20(3):377-3

For peer review only

For peer review only

BMJ Open

Measuring health related quality of life of care home residents, comparison of self-report with staff proxy responses for EQ-5D-5L and HowRu: Protocol for Assessing Proxy Reliability In Care home Outcome Testing

Journal:	*BMJ Open*
Manuscript ID	bmjopen-2018-022127.R1
Article Type:	Protocol
Date Submitted by the Author:	04-May-2018
Complete List of Authors:	Usman, Adeela; University of Nottingham, Division of Medical Sciences and Graduate Entry Medicine<br>Lewis, Sarah; University of Nottingham, Division of Epidemiology and Public Health<br>Hinsliff-Smith, Kathryn; University of Nottingham, Division of Medical Sciences and Graduate Entry Medicine<br>Long, Annabelle; University of Nottingham, Division of Medical Sciences and Graduate Entry Medicine<br>Housley, Gemma; East Midlands Academic Health Sciences Network<br>Jordan, Jake; University of Surrey, School of Economics<br>Gage, Heather; University of Surrey, School of Economics<br>Dening, Tom; University of Nottingham,<br>Gladman, John; University of Nottingham, Division of Rehabilitation and Ageing<br>Gordon, Adam; University of Nottingham, Division of Medical Sciences and Graduate Entry Medicine; City, University of London, School of Health Sciences
**Primary Subject Heading**:	Geriatric medicine
Secondary Subject Heading:	General practice / Family practice, Health economics
Keywords:	Quality of Life, Homes for the Aged, Frailty

SCHOLARONE™
Manuscripts

For peer review only - http://bmjopen.bmj.com/site/about/guidelines.xhtml

Measuring health related quality of life of care home residents, comparison of self-report with staff proxy responses for EQ-5D-5L and HowRu: Protocol for Assessing Proxy Reliability In Care home Outcome Testing

Adeela Usman [1]
Sarah Lewis [2]
Kathryn Hinsliff-Smith [1]
Annabelle Long [1]
Gemma Housley [3]
Jake Jordan [4]
Heather Gage [4]
Tom Dening [5]
John R F Gladman [6, 7]
Adam L Gordon [1, 8]

1. Division of Medical Sciences and Graduate Entry Medicine, School of Medicine, University of Nottingham, UK.
2. Division of Epidemiology and Public Health, School of Medicine, University of Nottingham, UK.
3. East Midlands East Midlands Academic Health Science Network, Nottingham, UK.
4. School of Economics, University of Surrey, UK.
5. Institute of Mental Health, University of Nottingham, UK.
6. Division of Rehabilitation and Ageing, University of Nottingham, UK
7. East Midlands Collaboration for Leadership in Applied Health Research and Care, UK
8. School of Health Sciences, City, University of London, UK.

Corresponding Author: Adam Gordon, Division of Medical Sciences and Graduate Entry Medicine, University of Nottingham, Room 4113, Derby Medical School, Derby. DE22 3NE.
Email: adam.gordon@nottingham.ac.uk; Tel: 01332 724668; Fax: 01332 724697

Word Count: 2793 words

For peer review only

For peer review only - http://bmjopen.bmj.com/site/about/guidelines.xhtml

Abstract

Introduction

Research into interventions to improve health and wellbeing for older people living in care homes is increasingly common. Health-Related Quality of Life (HRQoL) is frequently used as an outcome measure but collecting both self-reported and proxy HRQoL measures is challenging in this setting. This study will investigate the reliability of UK care home staff as proxy respondents for the EQ-5D-5L and HowRu measures.

Methods and Analysis

This is a prospective cohort study of a sub-population of care home residents recruited to the larger **P**roactive H**e**althc**a**re for Older People in **C**are **H**omes (PEACH) study. It will recruit residents ≥ 60 years across 24 care homes and not receiving short stay or respite care. The sample size is 160 participants. Resident and care home staff proxy EQ-5D-5L and HowRu responses will be collected monthly for three months. Weighted kappa statistics and intraclass correlation adjusted for clustering at the care home level will be used to measure agreement between resident and proxy responses. The extent to which staff variables (gender, age group, length of time caring, role, how well they know the resident, length of time working in care homes and in specialist gerontological practice) influence the level of agreement between self-reported and proxy responses will be considered using a multilevel mixed-effect regression model.

Ethics and Dissemination

The PEACH study protocol was reviewed by the UK Health Research Authority and University of Nottingham Research Ethics Committee and was determined to be a service development project. We will publish this study in a peer-reviewed journal with international readership and disseminate it through relevant national stakeholder networks and specialist societies.

For peer review only

For peer review only - http://bmjopen.bmj.com/site/about/guidelines.xhtml

Introduction

Long term care facilities in the UK are called care homes, and are classified as either care homes with or without nursing based upon the availability of registered nurses on-site (1). The types of residents cared for in both classifications of facility are similar and all UK care homes are included in the international consensus definition of a nursing home (2). Around 425,000 people live in care homes in the UK (3) with most residents requiring care due to disability from long-term conditions. The majority of residents are aged over 85, 75-80% of residents live with dementia (4), and over half of residents die within 12 months of admission to care home (5).

Improving the quality of care for older people in long-term care has become a focus of attention for health and social care providers, both within the UK and internationally (2), and an increasing number of evaluative research studies are being published that test the effectiveness and cost-effectiveness of interventions in this setting. Residents' quality of life (QoL) is frequently used as an outcome measure in these studies both to maintain a patient-centred focus and to facilitate health economic evaluation. The prevalent frailty and cognitive impairment in the care home population, however, means that collecting self-reported QoL measures from residents is challenging. As a response to this, proxy responses to QoL items have sometimes been used. For these a consultee, drawn from one of care home staff, or a relative or friend, answers questions on the resident's behalf. Using proxy respondents can be unreliable in care home settings. There may be lack of continuity of care home staff contact with individual residents due to shift working and staff turnover, and family and friends may not be well placed to judge QoL domains if they visit residents for only short periods (6).

The EurQoL questionnaire is a widely-used preference-based health-related quality of life (HRQoL) measure suitable for use in economic evaluations. The EQ-5D-5L version measures HRQoL across five domains (mobility, self-care, usual activities, pain, anxiety/depression) with the scale for each domain ranging from level 1 (no problems) to level 5 (extreme problems). The responses from the five domains are converted to QoL index scores (utilities) generated from a given country's general population (7). These index scores can be used to calculate quality adjusted life years (QALYs), which are a measure of the person's state of health – one QALY equates to one year in perfect health. QALYs are calculated using the area under the curve (8) defined by utility scores at the different assessment points over the study period. The cost per QALY gained from an intervention when compared to usual care is the chosen cost-utility measure for determining eligibility for public support of the intervention through the UK National Health Service (9).

The scale for the first version of EQ-5D had only three levels (EQ-5D-3L). EQ-5D-3L has been shown to have good construct validity for self-report (10), and has been used to measure QoL of older people living in their own homes and in care homes (11). The 5L version was developed subsequently to deal with identified issues with sensitivity and a ceiling effect on the EQ-5D-3L which limited its ability to discriminate between health states, particularly in those with higher quality of life (12). There is also an EQ-5D visual analogue scale (VAS) used to assess overall health status, ranging from 0 (worst imaginable) to 100 (best imaginable). VAS is recognised to have specific strengths and weaknesses(13) but is recommended to

For peer review only - http://bmjopen.bmj.com/site/about/guidelines.xhtml

be used routinely alongside the self-classification questionnaire by the EuroQoL group because of its usefulness in establishing global health status(14).

It is recognised that the EQ-5D, in all its forms, is limited by consequence of being a generic measure that fails to take account of the difference in what constitutes "quality of life" in a long-term care setting. It doesn't take account of shifts in emphasis about what constitutes wellbeing as residents enter long-term care, which means that social care related quality of life (SCRQoL) measures such as the Adult Social Care Outcomes Toolkit (ASCOT) may be preferable in this setting. A further critique has been that it fails to separate capability (what a resident is able to do) from preference (what a resident chooses to do under the circumstances), with the result that some authors have championed capability-based outcome measures, such as the ICEpop Capability Measure for Older People (ICECAP-O), in care homes (15-18). Best practice suggests that, if EQ-5D is used in this setting, it is used in combination with more specific instruments.

The R-outcome tool howRu has been specifically designed for use in long term care settings in order to address quality of life in a straightforward way that is practical with older people. HowRu is a Patient Recorded Outcome Measure (PROM) that records four variables (pain or discomfort, feeling low or worried, limitation in activities, and dependency on others) related to quality of life at a fixed point in time ("How are you doing today?") on a four-point scale (none, slight, quite a lot, extreme) (19, 20). The HowRu score is calculated by summing up the values for each domain to give a value on a 13-point scale ranging from 0 (worst) to 12 (best). The HowRu PROM was designed with older adults in mind, and may have a cogency and immediacy that improves upon some of the measurement uncertainty introduced by the relative abstraction of the questions included in highly validated general population indices such as EQ-5D-5L.

In a comparison with EQ-5D in patients attending a cardiovascular outpatient clinic, HowRu was reported to have better readability, higher completion rate, wider range of states used and smaller ceiling effect(17). No national tariffs exist to enable calculation of HowRu indices that would facilitate its use as a preference-based measure in cost-utility analysis. Understanding how and whether R outcomes correlate with EQ-5D scores in the care home setting is, however, helpful when considering additional information that can help triangulate our understanding of how interventions affect quality of life in this context. Knowing that HowRu correlates with EQ-5D may provide further justification for using it in clinical settings where broad judgements about impact on quality of life have to be made without the need for detailed cost-utility analysis.

Proxies have been used to capture EQ-5D-3L responses from people with dementia, although poor agreement between patient and proxy ratings has raised concerns (15,16) and differences between professional and family carer ratings have led to questions about the appropriate choice of proxy (16). In a comparison of clinicians and family carers as proxies, clinician responses had better construct validity in the more observable domains of mobility and self-care, and family carer responses had better construct validity in the less observable domains of usual activities and anxiety/depression (16). There is limited evidence, however, comparing self-reported and proxy responses to the EQ-5D-5L in care home populations (17). There is, in

For peer review only - http://bmjopen.bmj.com/site/about/guidelines.xhtml

particular, a paucity of data as to how it performs in UK care home populations. This is important because institutional care is structured differently between nations, with differing professional carer competencies, patterns of working and job roles. This means that carers in different countries will have differential exposure to residents and different competencies in terms of their ability to interpret residents' experiences, and a tool that works for professional proxy response in the US may not, therefore, work as well in the UK.

The HowRu, as a recently developed PROM, is yet to be fully evaluated for older people living in care homes (21). It is not known whether proxy responses in this setting may be useful in completing HowRU and there are no data on how HowRu correlates with EQ-5D in the care home populations.
This study seeks to fill these evidence gaps.

Aim

The Assessing Proxy Reliability In Care home Outcome Testing (APRICOT) study is a preparatory piece of work for the Proactive Healthcare for Older People Living in Care Homes (PEACH) study. It aims to examine the level of agreement between the responses to EQ-5D-5L and HowRu by care home staff and residents, and between EQ-5D-5L and HowRu as quality of life measures. Findings will assist in the interpretation of quality of life data gathered for the larger PEACH study.

Objectives

To determine the level of agreement between:

- Resident EQ-5D-5L and staff-proxy EQ-5D-5L responses
- Resident HowRu and staff-proxy HowRu responses
- Resident EQ-5D-5L and HowRu responses
- Proxy EQ-5D-5L and HowRu responses

Methods

Setting

24 care homes in the East Midlands area of England. These are long-term care institutions, housing predominantly older people with frailty who can no longer be cared for at home. Detailed descriptions of the UK care home sector and the residents living within it have been published elsewhere (4).

Brief description of the Proactive Healthcare in Older Peoples' Care Homes (PEACH) study

The PEACH intervention involves using Quality Improvement Collaboratives as a mechanism to encourage implementation of Comprehensive Geriatric Assessment (CGA) as a unifying framework for assessment and delivery of healthcare in UK care homes. CGA is widely recognised as a gold-standard way to deliver care for older people with frailty (22). The aim of PEACH is to improve quality of care and quality of life for care home residents. Outcome data quantifying healthcare resource use and resident level quality of life will be collected on a monthly basis to assess the impact of the intervention.

For peer review only - http://bmjopen.bmj.com/site/about/guidelines.xhtml

Two instruments are being used in PEACH to assess residents' quality of life, the EQ-5D-5L and HowRu. The rationale is that these reflect measurable differences in the patient experience that may translate, with some interpretation, into an understanding of how CGA influences quality of care and general wellbeing. APRICOT has been designed as a preparatory sub-study within PEACH to better enable interpretation of proxy EQ-5D-5L and HowRU responses collected as part of outcome measurement.

Participants

Care home recruitment for PEACH took place between October 2016 and January 2017, with individual resident recruitment from January 2017. A prospective cohort of a sub-population of residents will be included in the comparison of proxy and self-report measurement of EQ-5D-5L and HowRu in APRICOT. Residents included in the study will be those ≥ 60 years across 24 care homes and not receiving short stay or respite care .To have a better reflection of self-reported and proxy agreement in a care home setting we will include residents with and without mental capacity.Care home managers will provide guidance with regards to residents with and without capacity to participate. When managers are unsure, researchers will make judgements based on the framework for mental capacity outlined in the 2005 Mental Capacity Act for England and Wales (23) and in keeping with the recommendations of that Act for inclusion research, for residents that lack capacity to provide consent to participation an appropriate person will be consulted to make a decision with regards to participation in the study.

This study will be conducted in parallel to the main PEACH study. In addition to the routine collection of EQ-5D-5L and HowRu from residents recruited to PEACH, proxy responses to EQ-5D-5L and HowRu will be gathered from staff. We will include staff such as care home assistants, care home manager and registered nurses, who know the resident well. We will exclude staff employed in a supportive role, such as activity coordinators, since their orientation to supporting residents is more variable.

Data collection

Data from proxies will be collected on three consecutive months. Due to the high staff turnover amongst care home staff, and to enable the influence of carer characteristics on the level of agreement to be estimated, data on the carer will be gathered at each assessment. Repeated measures are required for the final analysis in the PEACH study for calculating costs per QALY gained (comparing the intervention with usual care condition) and understanding how the agreement changes at different time points is therefore of interest.

Staff proxies will be asked to consider the proxy-resident's perspective when completing the questionnaire using the following statement: "*Please rate how you (staff) think the resident will rate his/her own health-realted quality of life, if the resident was to communicate"* (24). Both self-reported and proxy EQ-5D will be completed on the same day to minimize any variations in responses.

The EQ-5D VAS will be used in the study in keeping with the recommendations of the EuroQoL group.

For peer review only - http://bmjopen.bmj.com/site/about/guidelines.xhtml

Primary analysis

An overall agreement between the self-reported and staff proxy responses on the domain levels of the EQ-5D-5L and HowRu will be computed. Weighted kappa statistic and Intra-class correlation (ICC) will be used to calculate the level of agreement for categorical and continuous outcomes respectively. All reliability indices will be calculated at the domain levels and overall index scores/QALYs for the EQ-5D-5L. To calculate the EQ-5D-5L index scores, responses from the descriptive system will be transformed into index scores derived from the UK general population. This will be done using the crosswalk value set (25), which maps the 5L descriptive system data onto the 3L valuation.

Weighted kappa helps to distinguish between small and large difference in agreement ratings assigned to the different levels of each domain but equal importance is given to disagreement (26, 27). The weighting for kappa will be done using linear weight - this assigns the same importance to the difference between any two categories within the response scale (28). The confidence interval for the weighted kappa will be calculated by bootstrapping in Stata 15 (Statcorp, LLC, 2015) with 1000 replications. This will be done for each time point.

The kappa statistic ranges from -1 to 1, and the strength of the agreement will be interpreted with regards to published guidelines (29) with agreement being:

- Poor, if kappa ≤ 0.00
- Slight, if kappa = 0.00 to 0.20
- Fair, if kappa = 0.21 to 0.40
- Moderate, if kappa = 0.41 to 0.60
- Substantial, if kappa = 0.61 to 0.80
- Almost perfect, if kappa ≥ 0.80

Unadjusted ICC will be calculated using two-way mixed effect ANOVA model to examine the level of agreement between the self-reported and proxy responses for the EQ-5D-VAS, EQ-5D index scores and total QALYs. ANOVA models are reported to be robust to deviations in normality and have been used in other quality of life agreement studies (16,30).The Bland-Altman graph (plotting the mean difference between the EQ-5D-5L-S (self-report) and EQ-5D-5L-P (proxy) against the mean of the two measures) will be constructed to supplement the ICC .

Analysis will be done at each time point for Kappa and ICC. However, a single ICC value for QALYs will be calculated for individuals with data on all three consecutive months as this will be used in practice in the PEACH study, where analysis will be done on consecutive measures made over time.

To allow for comparability of the level of agreement at the domain and index score level; the same benchmarks used for kappa will be used for the ICC.

Clustering

Clustering will be adjusted for because the calculation of kappa and ICC assumes independence of observations. In our study, clustering could occur at three levels at each time point. Firstly, at the care home level, where residents within the same care home have similar characteristics and are different from those in other care homes. Secondly, at the individual level, where responses are clustered within each resident

For peer review only - http://bmjopen.bmj.com/site/about/guidelines.xhtml

and lastly, at staff level, where staff members within a care home responds on behalf of multiple residents.

The ICC value will be calculated allowing for clustering using a nested two-way mixed effect model calculated by fitting a 2 level random effect model with a random effect for care home and individuals.

A cluster adjusted kappa will be calculated using a variance formula. This will include calculating kappa and its variance for each care home, then estimating the within cluster variance σ_{ω}^2 using the average of the individual variances, and between cluster variance σ_b^2 using the variance of the individual kappa. Using these estimates the clustered Kappa and its variance will be calculated using the formulas below:

$$\omega_i = \frac{n_i}{(1+(n_i-1)\rho_k)}$$

Where
n_i = Size of cluster i
ρ = Intra-cluster correlation coefficient for Kappa

$$\rho_k = \frac{\sigma_b^2}{(\sigma_{\omega}^2 + \sigma_b^2)}$$

Variance of the cluster adjusted kappa will be obtained using the equation below (24):

$$\sigma^2_{clustered\ k} = \frac{\sum_{i=1}^{k} \omega_i^2 \left(\sigma_b^2 + \frac{\sigma_{\omega}^2}{n_i}\right)}{\left(\sum_{i=1}^{k} \omega_i\right)^2}$$

The clustered kappa and its variance will then be divided by the square root of the number of individuals to get the standard error. The 95% confidence interval will be calculated using this standard error.

Cluster adjusted kappa (EQ-5D-5L and HowRu domain levels) and ICC (EQ-VAS and EQ-5D index scores) will be reported for each time point. However, QALYs will be presented over time, three months, similar to how it will be calculated in the PEACH study.

Sample size calculation

We need a sample size of 160 residents assuming a kappa of 0.145 and a confidence level width of 0.153 derived from a previous study (16), given that 50% of residents will have any problems.

Secondary analyses

The effect of age, sex and length of stay in care home (for residents), length time working in care of older people/care homes and role/rank (for staff) at baseline on the difference between staff and proxy EQ-5D-5L-S scores will be analysed using a multilevel mixed effect regression model.

For peer review only - http://bmjopen.bmj.com/site/about/guidelines.xhtml

To investigate the reliability of using HowRu as a quality of life measure in the care home population compared with EQ-5D-5L, we will assess agreement between these indices using weighted kappa statistics. This will involve testing the level of agreement between domains with similar construct on both scales (31) as shown in table 1.

Table 1 - Planned pair-wise alignment of HowRU and EQ-5D-5L domains for agreement analysis using kappa

HowRu domains	EQ-5D-5L domains
Pain or discomfort	Pain/discomfort
Feeling low or worried	Anxiety/depression
Limited in what you can do	Mobility<br>Self-care<br>Usual activities
Dependent on others	Mobility<br>Self-care<br>Usual activities

Patient and Public Involvement

The APRICOT and PEACH studies were developed and designed in discussion with both Care Home Sector and Patient and Public Involvement (PPI) representatives. The initial research proposal and protocol was presented, prior to submission for funding, to the Dementia and Frail Older Person's PPI group hosted in the Division of Rehabilitation and Ageing, University of Nottingham. Amendments were made to the proposal and protocol in light of their feedback. The PEACH study team includes one PPI and two care home sector representatives who are present at all study management meetings, with oversight for the APRICOT sub-study. We keep all participating care homes working with PEACH updated through quarterly newsletters which will include dissemination of our findings in lay terms as these become available.

Strengths and Limitations of this study

- To our knowledge, this is the first study to investigate the reliability of staff proxy using the EQ-5D-5L and HowRu, and the reliability of HowRu as a quality of life measure in a UK care home setting.
- The scales will be administered to residents and staff on the same day, thus reducing any variations that will affect the level of agreement.
- Analysing the characteristics of staff respondents will help generate insights into how proxies might better be selected or prepared to maximise the validity of proxy responses.
- The use of multiple time points will help increase the sample size and account for the influence of staff turnover on the quality of proxy-response.
- Working with residents with mental capacity will optimise their responses as a gold standard against which proxies can be compared. The way in which staff interpret quality of life in this more communicative and able group may, however, be different to the way in which the interpret quality of life in advanced dementia.

For peer review only - http://bmjopen.bmj.com/site/about/guidelines.xhtml

Ethics and dissemination

This study is part of preparatory work for the larger PEACH study. PEACH was reviewed by both the UK Health Research Authority and the University of Nottingham Research Ethics Committee and determined by both to be a service development and evaluation project. We will obtain informed consent from residents who have mental capacity and use a consultee when residents lack mental capacity. The PEACH study protocol has been reviewed as part of good governance by the Nottinghamshire Healthcare Foundation Trust. We aim to publish this study in a peer-reviewed journal with international readership and disseminate it further using relevant national stakeholder networks and specialist societies.

Funding

This work has been conducted as part of the Proactive Healthcare of Older People in Care Homes (PEACH) study, supported by the Dunhill Medical Trust, award number FOP1/0115.

Contributors

All authors meet the ICJME criteria for authorship. AU, SL, KHS, AL, GH, JJ, HG, TD, JRFG and ALG conceived the study at a PEACH study management meeting and specified the aims and objectives. AU, SL and ALG produced the initial draft of the protocol, with KHS, AL, GH, JJ, HG, TD and JRFG contributing to subsequent redrafts. AU and SL led on aspects of statistical design. JJ and HG provided specialist health economics input. AU, SL, KHS, AL, GH, JJ, HG, TD, JRFG and ALG all reviewed the final manuscript and approved it prior to submission.

Competing interests

None declared.

Acknowledgements

The authors would like to acknowledge the broader PEACH study team: Mr Zimran Alam, Ms Anita Astle, Professor Tony Avery, Dr Jaydip Banerjee, Professor Clive Bowman, Dr Neil H Chadborn, Mr Michael Crossley, Dr Reena Devi, Professor Claire Goodman, Professor Pip Logan, Professor Finbarr Martin, Professor Julienne Meyer, Dr Dominick Shaw, Professor David Stott and Dr Maria Zubair.

References

1. Aspden T, Bradshaw SA, Playford ED, Riazi A. Quality-of-life measures for use within care homes: a systematic review of their measurement properties. Age and Ageing. 2014; 43(5):596-603.
2. Sanford AM, Orrell M, Tolson D, Abbatecola AM, Arai H, Bauer JM, et al. An International Definition for “Nursing Home”. Journal of the American Medical Directors Association. 2015;16(3):181-184.
3. Laing & Buisson. Care of the Elderly People Market Survey. London: Laing and Buisson 2010; 2009.
4. Gordon AL, Franklin M, Bradshaw L, Logan P, Elliott R, Gladman JRF. Health status of UK care home residents: a cohort study. Age and Ageing. 2014;43(1):97-103.

For peer review only - http://bmjopen.bmj.com/site/about/guidelines.xhtml

5. Kinley J, Hockley J, Stone L, Dewey M, Hansford P, Stewart R, et al. The provision of care for residents dying in UK nursing care homes. Age and Ageing. 2014;43(3):375-379.
6. Kane RL, Kane RA, Bershadsky B, Degenholtz H, Kling K, Totten A, et al. Proxy sources for information on nursing home residents' quality of life. The journals of gerontology Series B, Psychological sciences and social sciences. 2005;60(6):S318-s325.
7. Dolan P, Gudex C, Kind P, Williams A. A social tariff for EuroQol: Results from a UK general population survey. The University of York Centre for Health Economics; 1995. [Accessed 7 December 2017]. Discussion Paper 138. Available from: http://www.york.ac.uk/che/pdf/DP138.pdf.
8. Whitehead SJ, Ali S. Health outcomes in economic evaluation: the QALY and utilities. Br Med Bull. 2010;96(1):5–21.
9. McCabe C, Claxton K, Culyer AJ. The NICE Cost-EffectivenessThreshold. Pharmacoeconomics. 2008;26(9):733-744.
10. Janssen MF, Simon Pickard A, Golicki D, Gudex C, Niewada M et al. Measurement properties of the EQ-5D-5L compared to the EQ-5D-3L across eight patient groups: a multi-country study. Qual Life Res. 2013; 22(7): 1717-27
11. Borowiak E, Kostka T. Predictors of quality of life in older people living at home and in institutions. Aging Clinical and Experimental Research. 2004;16(3):212-220.
12. Devlin NJ, Brooks R. EQ-5D and the EuroQol Group: Past, Present and Future. Appl Health Econ Health Policy. 2017;15(2):127–37.
13. Parkin D, Devlin N. Is there a case for using visual analogue scale valuations in cost-utility analysis? Health Econ [Internet]. 2006 Jul 1 [cited 2018 Apr 25];15(7):653–64. Available from: http://doi.wiley.com/10.1002/hec.1086
14. Feng Y, Parkin D, Devlin NJ. Assessing the performance of the EQ-VAS in the NHS PROMs programme. Qual Life Res. 2014; 23(3):977–89.
15. Hounsome N, Orrell M, Edwards RT. EQ-5D as a Quality of Life Measure in People with Dementia and Their Carers: Evidence and Key Issues. Value in Health. 2011;14(2):390-399.
16. Devine A, Taylor SJC, Spencer A, Diaz-Ordaz K, Eldridge S, Underwood M. The agreement between proxy and self-completed EQ-5D for care home residents was better for index scores than individual domains. Journal of Clinical Epidemiology. 2014;67(9):1035-1043.
17. Bulamu NB, Kaambwa B, Ratcliffe J. A systematic review of instruments for measuring outcomes in economic evaluation within aged care. Health and Quality of Life Outcomes. 2015;13(1).
18. van Leeuwen KM, Bosmans JE, Jansen AP, Hoogendijk EO, van Tulder MW, van der Horst HE, et al. Comparing Measurement Properties of the EQ-5D-3L, ICECAP-O, and ASCOT in Frail Older Adults. Value in health : the Journal of the International Society for Pharmacoeconomics and Outcomes Research. 2015;18(1):35-43.
19. Benson T, Williams DH, Potts HWW. Performance of EQ-5D, howRu and Oxford hip & knee scores in assessing the outcome of hip and knee replacements. BMC Health Services Research. 2016;16(1).
20. Benson T, Potts HWW, Whatling JM, Patterson D. Comparison of howRU and EQ-5D measures of health-related quality of life in an outpatient clinic. Inform Prim Care. 2013;21(1):12-17.
21. Hendriks SH, Rutgers J, van Dijk PR, Groenier KH, Bilo HJG, Kleefstra N, et al. Validation of the howRu and howRwe questionnaires at the individual patient level. BMC Health Services Research. 2015;15(1).
22. Welsh TJ, Gordon AL, Gladman JR. Comprehensive geriatric assessment - a guide for the non-specialist. *Int J Clin Pract*. 2014. 68(3): 290–293

For peer review only

For peer review only - http://bmjopen.bmj.com/site/about/guidelines.xhtml

23. Dobson C. Conducting research with people not having the capacity to consent to their participation. A practical guide for researchers [Internet]. Leicester, UK: The British Psychological Society; 2017 [cited 1 December 2017]. Available from: https://pdfs.semanticscholar.org/f97c/5a3d8605e24eaaf965b64e42c690d42a6182.pdf

24. Selai C, Pickard S, Johnston JA. The collection of EuroQol EQ-5D data via proxies. EQ Plenary Meeting in York: September, 2002

25. van Hout B, Janssen MF, Feng Y-S, Kohlmann T, Busschbach J, Golicki D, et al. Interim Scoring for the EQ-5D-5L: Mapping the EQ-5D-5L to EQ-5D-3L Value Sets. Value Heal.;15(5):708–15.

26. Vanbelle S, Albert A. A note on the linearly weighted kappa coefficient for ordinal scales. Statistical Methodology. 2009; 6(2):157-163.

27. Brenner H, Kliebsch U. Dependence of Weighted Kappa Coefficients on the Number of Categories. Epidemiology. 1996;7(2):199-202.

28. Cicchetti DV, Allison T. A New Procedure for Assessing Reliability of Scoring EEG Sleep Recordings. American Journal of EEG Technology. 1971;11(3):101-110.

29. Landis JR, Koch GG. The measurement of observer agreement for categorical data. Biometrics. 1977;33(1):159.

30. Harwell MR, Rubinstein EN, Hayes WS, Olds CC. Summarizing Monte Carlo Results in Methodological Research: The One- and Two-Factor Fixed Effects ANOVA Cases. J Educ Stat. 1992 Dec 23;17(4):315–39.

31. Kerry SM, Bland JM. Unequal cluster sizes for trials in English and Welsh general practice: implications for sample size calculations. Statistics in Medicine. 2001;20(3):377-

For peer review only

For peer review only - http://bmjopen.bmj.com/site/about/guidelines.xhtml

For peer review only

For peer review only - http://bmjopen.bmj.com/site/about/guidelines.xhtml

BMJ Open

Measuring health related quality of life of care home residents, comparison of self-report with staff proxy responses for EQ-5D-5L and HowRu: Protocol for Assessing Proxy Reliability In Care home Outcome Testing

Journal:	*BMJ Open*
Manuscript ID	bmjopen-2018-022127.R2
Article Type:	Protocol
Date Submitted by the Author:	20-Jun-2018

Complete List of Authors:	Usman, Adeela; University of Nottingham, Division of Medical Sciences and Graduate Entry Medicine<br>Lewis, Sarah; University of Nottingham, Division of Epidemiology and Public Health<br>Hinsliff-Smith, Kathryn; University of Nottingham, Division of Medical Sciences and Graduate Entry Medicine<br>Long, Annabelle; University of Nottingham, Division of Medical Sciences and Graduate Entry Medicine<br>Housley, Gemma; East Midlands Academic Health Sciences Network<br>Jordan, Jake; University of Surrey, School of Economics<br>Gage, Heather; University of Surrey, School of Economics<br>Dening, Tom; University of Nottingham,<br>Gladman, John; University of Nottingham, Division of Rehabilitation and Ageing<br>Gordon, Adam; University of Nottingham, Division of Medical Sciences and Graduate Entry Medicine; City, University of London, School of Health Sciences

**Primary Subject Heading**:	Geriatric medicine
Secondary Subject Heading:	General practice / Family practice, Health economics
Keywords:	Quality of Life, Homes for the Aged, Frailty

SCHOLARONE™ Manuscripts

Measuring health related quality of life of care home residents, comparison of self-report with staff proxy responses for EQ-5D-5L and HowRu: Protocol for Assessing Proxy Reliability In Care home Outcome Testing

Adeela Usman [1]
Sarah Lewis [2]
Kathryn Hinsliff-Smith [1]
Annabelle Long [1]
Gemma Housley [3]
Jake Jordan [4]
Heather Gage [4]
Tom Dening [5]
John R F Gladman [6, 7]
Adam L Gordon [1, 8]

1. Division of Medical Sciences and Graduate Entry Medicine, School of Medicine, University of Nottingham, UK.
2. Division of Epidemiology and Public Health, School of Medicine, University of Nottingham, UK.
3. East Midlands East Midlands Academic Health Science Network, Nottingham, UK.
4. School of Economics, University of Surrey, UK.
5. Institute of Mental Health, University of Nottingham, UK.
6. Division of Rehabilitation and Ageing, University of Nottingham, UK
7. East Midlands Collaboration for Leadership in Applied Health Research and Care, UK
8. School of Health Sciences, City, University of London, UK.

Corresponding Author: Adam Gordon, Division of Medical Sciences and Graduate Entry Medicine, University of Nottingham, Room 4113, Derby Medical School, Derby. DE22 3NE.
Email: adam.gordon@nottingham.ac.uk; Tel: 01332 724668; Fax: 01332 724697

Word Count: 2793 words

For peer review only

For peer review only - http://bmjopen.bmj.com/site/about/guidelines.xhtml

Abstract

Introduction

Research into interventions to improve health and wellbeing for older people living in care homes is increasingly common. Health-Related Quality of Life (HRQoL) is frequently used as an outcome measure but collecting both self-reported and proxy HRQoL measures is challenging in this setting. This study will investigate the reliability of UK care home staff as proxy respondents for the EQ-5D-5L and HowRu measures.

Methods and Analysis

This is a prospective cohort study of a sub-population of care home residents recruited to the larger **P**roactive H**e**althc**a**re for Older People in **C**are **H**omes (PEACH) study. It will recruit residents ≥ 60 years across 24 care homes and not receiving short stay or respite care. The sample size is 160 participants. Resident and care home staff proxy EQ-5D-5L and HowRu responses will be collected monthly for three months. Weighted kappa statistics and intraclass correlation adjusted for clustering at the care home level will be used to measure agreement between resident and proxy responses. The extent to which staff variables (gender, age group, length of time caring, role, how well they know the resident, length of time working in care homes and in specialist gerontological practice) influence the level of agreement between self-reported and proxy responses will be considered using a multilevel mixed-effect regression model.

Ethics and Dissemination

The PEACH study protocol was reviewed by the UK Health Research Authority and University of Nottingham Research Ethics Committee and was determined to be a service development project. We will publish this study in a peer-reviewed journal with international readership and disseminate it through relevant national stakeholder networks and specialist societies.

For peer review only - http://bmjopen.bmj.com/site/about/guidelines.xhtml

Introduction

Long term care facilities in the UK are called care homes, and are classified as either care homes with or without nursing based upon the availability of registered nurses on-site (1). The types of residents cared for in both classifications of facility are similar and all UK care homes are included in the international consensus definition of a nursing home (2). Around 425,000 people live in care homes in the UK (3) with most residents requiring care due to disability from long-term conditions. The majority of residents are aged over 85, 75-80% of residents live with dementia (4), and over half of residents die within 12 months of admission to care home (5).

Improving the quality of care for older people in long-term care has become a focus of attention for health and social care providers, both within the UK and internationally (2), and an increasing number of evaluative research studies are being published that test the effectiveness and cost-effectiveness of interventions in this setting. Residents' quality of life (QoL) is frequently used as an outcome measure in these studies both to maintain a patient-centred focus and to facilitate health economic evaluation. The prevalent frailty and cognitive impairment in the care home population, however, means that collecting self-reported QoL measures from residents is challenging. As a response to this, proxy responses to QoL items have sometimes been used. For these a consultee, drawn from one of care home staff, or a relative or friend, answers questions on the resident's behalf. Using proxy respondents can be unreliable in care home settings. There may be lack of continuity of care home staff contact with individual residents due to shift working and staff turnover, and family and friends may not be well placed to judge QoL domains if they visit residents for only short periods (6).

The EurQoL questionnaire is a widely-used preference-based health-related quality of life (HRQoL) measure suitable for use in economic evaluations. The EQ-5D-5L version measures HRQoL across five domains (mobility, self-care, usual activities, pain, anxiety/depression) with the scale for each domain ranging from level 1 (no problems) to level 5 (extreme problems). The responses from the five domains are converted to QoL index scores (utilities) generated from a given country's general population (7). These index scores can be used to calculate quality adjusted life years (QALYs), which are a measure of the person's state of health – one QALY equates to one year in perfect health. QALYs are calculated using the area under the curve (8) defined by utility scores at the different assessment points over the study period. The cost per QALY gained from an intervention when compared to usual care is the chosen cost-utility measure for determining eligibility for public support of the intervention through the UK National Health Service (9).

The scale for the first version of EQ-5D had only three levels (EQ-5D-3L). EQ-5D-3L has been shown to have good construct validity for self-report (10), and has been used to measure QoL of older people living in their own homes and in care homes (11). The 5L version was developed subsequently to deal with identified issues with sensitivity and a ceiling effect on the EQ-5D-3L which limited its ability to discriminate between health states, particularly in those with higher quality of life (12). There is also an EQ-5D visual analogue scale (VAS) used to assess overall health status, ranging from 0 (worst imaginable) to 100 (best imaginable). VAS is recognised to have specific strengths and weaknesses(13) but is recommended to

For peer review only - http://bmjopen.bmj.com/site/about/guidelines.xhtml

be used routinely alongside the self-classification questionnaire by the EuroQoL group because of its usefulness in establishing global health status(14).

It is recognised that the EQ-5D, in all its forms, is limited by consequence of being a generic measure that fails to take account of the difference in what constitutes "quality of life" in a long-term care setting. It doesn't take account of shifts in emphasis about what constitutes wellbeing as residents enter long-term care, which means that social care related quality of life (SCRQoL) measures such as the Adult Social Care Outcomes Toolkit (ASCOT) may be preferable in this setting (15-16). A further critique has been that it fails to separate capability (what a resident is able to do) from preference (what a resident chooses to do under the circumstances), with the result that some authors have championed capability-based outcome measures, such as the ICEpop Capability Measure for Older People (ICECAP-O), in care homes (17-18). Best practice suggests that, if EQ-5D is used in this setting, it is used in combination with more specific instruments.

The R-outcome tool howRu has been specifically designed for use in long term care settings in order to address quality of life in a straightforward way that is practical with older people. HowRu is a Patient Recorded Outcome Measure (PROM) that records four variables (pain or discomfort, feeling low or worried, limitation in activities, and dependency on others) related to quality of life at a fixed point in time ("How are you doing today?") on a four-point scale (none, slight, quite a lot, extreme) (19, 20). The HowRu score is calculated by summing up the values for each domain to give a value on a 13-point scale ranging from 0 (worst) to 12 (best). The HowRu PROM was designed with older adults in mind (19,20), and may have a cogency and immediacy that improves upon some of the measurement uncertainty introduced by the relative abstraction of the questions included in highly validated general population indices such as EQ-5D-5L.

In a comparison with EQ-5D in patients attending a cardiovascular outpatient clinic, HowRu was reported to have better readability, higher completion rate, wider range of states used and smaller ceiling effect(17). No national tariffs exist to enable calculation of HowRu indices that would facilitate its use as a preference-based measure in cost-utility analysis. Understanding how and whether R outcomes correlate with EQ-5D scores in the care home setting is, however, helpful when considering additional information that can help triangulate our understanding of how interventions affect quality of life in this context. Knowing that HowRu correlates with EQ-5D may provide further justification for using it in clinical settings where broad judgements about impact on quality of life have to be made without the need for detailed cost-utility analysis.

Proxies have been used to capture EQ-5D-3L responses from people with dementia, although poor agreement between patient and proxy ratings has raised concerns (15,16) and differences between professional and family carer ratings have led to questions about the appropriate choice of proxy (16). In a comparison of clinicians and family carers as proxies, clinician responses had better construct validity in the more observable domains of mobility and self-care, and family carer responses had better construct validity in the less observable domains of usual activities and anxiety/depression (16). There is limited evidence, however, comparing self-reported and proxy responses to the EQ-5D-5L in care home populations (17). There is, in

For peer review only - http://bmjopen.bmj.com/site/about/guidelines.xhtml

particular, a paucity of data as to how it performs in UK care home populations. This is important because institutional care is structured differently between nations, with differing professional carer competencies, patterns of working and job roles. This means that carers in different countries will have differential exposure to residents and different competencies in terms of their ability to interpret residents' experiences, and a tool that works for professional proxy response in the US may not, therefore, work as well in the UK.

The HowRu, as a recently developed PROM, is yet to be fully evaluated for older people living in care homes (21). It is not known whether proxy responses in this setting may be useful in completing HowRU and there are no data on how HowRu correlates with EQ-5D in the care home populations.
This study seeks to fill these evidence gaps.

Aim

The Assessing Proxy Reliability In Care home Outcome Testing (APRICOT) study is a preparatory piece of work for the Proactive Healthcare for Older People Living in Care Homes (PEACH) study. It aims to examine the level of agreement between the responses to EQ-5D-5L and HowRu by care home staff and residents, and between EQ-5D-5L and HowRu as quality of life measures. Findings will assist in the interpretation of quality of life data gathered for the larger PEACH study.

Objectives

To determine the level of agreement between:

- Resident EQ-5D-5L and staff-proxy EQ-5D-5L responses
- Resident HowRu and staff-proxy HowRu responses
- Resident EQ-5D-5L and HowRu responses
- Proxy EQ-5D-5L and HowRu responses

Methods

Setting

24 care homes in the East Midlands area of England. These are long-term care institutions, housing predominantly older people with frailty who can no longer be cared for at home. Detailed descriptions of the UK care home sector and the residents living within it have been published elsewhere (4).

Brief description of the Proactive Healthcare in Older Peoples' Care Homes (PEACH) study

The PEACH intervention involves using Quality Improvement Collaboratives as a mechanism to encourage implementation of Comprehensive Geriatric Assessment (CGA) as a unifying framework for assessment and delivery of healthcare in UK care homes. CGA is widely recognised as a gold-standard way to deliver care for older people with frailty (22). The aim of PEACH is to improve quality of care and quality of life for care home residents. Outcome data quantifying healthcare resource use and resident level quality of life will be collected on a monthly basis to assess the impact of the intervention.

For peer review only

For peer review only - http://bmjopen.bmj.com/site/about/guidelines.xhtml

Two instruments are being used in PEACH to assess residents' quality of life, the EQ-5D-5L and HowRu. The rationale is that these reflect measurable differences in the patient experience that may translate, with some interpretation, into an understanding of how CGA influences quality of care and general wellbeing. APRICOT has been designed as a preparatory sub-study within PEACH to better enable interpretation of proxy EQ-5D-5L and HowRU responses collected as part of outcome measurement.

Participants

Care home recruitment for PEACH took place between October 2016 and January 2017, with individual resident recruitment from January 2017. A prospective cohort of a sub-population of residents will be included in the comparison of proxy and self-report measurement of EQ-5D-5L and HowRu in APRICOT. Residents included in the study will be those ≥ 60 years across 24 care homes and not receiving short stay or respite care .To have a better reflection of self-reported and proxy agreement in a care home setting we will include residents with and without mental capacity.Care home managers will provide guidance with regards to residents with and without capacity to participate. When managers are unsure, researchers will make judgements based on the framework for mental capacity outlined in the 2005 Mental Capacity Act for England and Wales (23) and in keeping with the recommendations of that Act for inclusion research, for residents that lack capacity to provide consent to participation an appropriate person will be consulted to make a decision with regards to participation in the study.

This study will be conducted in parallel to the main PEACH study. In addition to the routine collection of EQ-5D-5L and HowRu from residents recruited to PEACH, proxy responses to EQ-5D-5L and HowRu will be gathered from staff. We will include staff such as care home assistants, care home manager and registered nurses, who know the resident well. We will exclude staff employed in a supportive role, such as activity coordinators, since their orientation to supporting residents is more variable.

Data collection

Data from proxies will be collected on three consecutive months. Due to the high staff turnover amongst care home staff, and to enable the influence of carer characteristics on the level of agreement to be estimated, data on the carer will be gathered at each assessment. Repeated measures are required for the final analysis in the PEACH study for calculating costs per QALY gained (comparing the intervention with usual care condition) and understanding how the agreement changes at different time points is therefore of interest.

Staff proxies will be asked to consider the proxy-resident's perspective when completing the questionnaire using the following statement: "*Please rate how you (staff) think the resident will rate his/her own health-realted quality of life, if the resident was to communicate"* (24). Both self-reported and proxy EQ-5D will be completed on the same day to minimize any variations in responses.

The EQ-5D VAS will be used in the study in keeping with the recommendations of the EuroQoL group.

For peer review only - http://bmjopen.bmj.com/site/about/guidelines.xhtml

Primary analysis

An overall agreement between the self-reported and staff proxy responses on the domain levels of the EQ-5D-5L and HowRu will be computed. Weighted kappa statistic and Intra-class correlation (ICC) will be used to calculate the level of agreement for categorical and continuous outcomes respectively. All reliability indices will be calculated at the domain levels and overall index scores/QALYs for the EQ-5D-5L. To calculate the EQ-5D-5L index scores, responses from the descriptive system will be transformed into index scores derived from the UK general population. This will be done using the crosswalk value set (25), which maps the 5L descriptive system data onto the 3L valuation.

Weighted kappa helps to distinguish between small and large difference in agreement ratings assigned to the different levels of each domain but equal importance is given to disagreement (26, 27). The weighting for kappa will be done using linear weight - this assigns the same importance to the difference between any two categories within the response scale (28). The confidence interval for the weighted kappa will be calculated by bootstrapping in Stata 15 (Statcorp, LLC, 2015) with 1000 replications. This will be done for each time point.

The kappa statistic ranges from -1 to 1, and the strength of the agreement will be interpreted with regards to published guidelines (29) with agreement being:

- Poor, if kappa ≤ 0.00
- Slight, if kappa = 0.00 to 0.20
- Fair, if kappa = 0.21 to 0.40
- Moderate, if kappa = 0.41 to 0.60
- Substantial, if kappa = 0.61 to 0.80
- Almost perfect, if kappa ≥ 0.80

Unadjusted ICC will be calculated using two-way mixed effect ANOVA model to examine the level of agreement between the self-reported and proxy responses for the EQ-5D-VAS, EQ-5D index scores and total QALYs. ANOVA models are reported to be robust to deviations in normality and have been used in other quality of life agreement studies (16,30).The Bland-Altman graph (plotting the mean difference between the EQ-5D-5L-S (self-report) and EQ-5D-5L-P (proxy) against the mean of the two measures) will be constructed to supplement the ICC .

Analysis will be done at each time point for Kappa and ICC. However, a single ICC value for QALYs will be calculated for individuals with data on all three consecutive months as this will be used in practice in the PEACH study, where analysis will be done on consecutive measures made over time.

To allow for comparability of the level of agreement at the domain and index score level; the same benchmarks used for kappa will be used for the ICC.

Clustering

Clustering will be adjusted for because the calculation of kappa and ICC assumes independence of observations. In our study, clustering could occur at three levels at each time point. Firstly, at the care home level, where residents within the same care home have similar characteristics and are different from those in other care homes. Secondly, at the individual level, where responses are clustered within each resident

For peer review only - http://bmjopen.bmj.com/site/about/guidelines.xhtml

and lastly, at staff level, where staff members within a care home responds on behalf of multiple residents.

The ICC value will be calculated allowing for clustering using a nested two-way mixed effect model calculated by fitting a 2 level random effect model with a random effect for care home and individuals.

A cluster adjusted kappa will be calculated using a variance formula. This will include calculating kappa and its variance for each care home, then estimating the within cluster variance σ_ω^2using the average of the individual variances, and between cluster variance σ_b^2using the variance of the individual kappa. Using these estimates the clustered Kappa and its variance will be calculated using the formulas below:

$$\omega_i = \frac{n_i}{(1+(n_i-1)\rho_k)}$$

Where
n_i = Size of cluster i
ρ = Intra-cluster correlation coefficient for Kappa

$$\rho_k = \frac{\sigma_b^2}{(\sigma_\omega^2 + \sigma_b^2)}$$

Variance of the cluster adjusted kappa will be obtained using the equation below (24):

$$\sigma^2_{clustered\ k} = \frac{\sum_{i=1}^{k} \omega_i^2 \left(\sigma_b^2 + \frac{\sigma_\omega^2}{n_i}\right)}{\left(\sum_{i=1}^{k} \omega_i\right)^2}$$

The clustered kappa and its variance will then be divided by the square root of the number of individuals to get the standard error. The 95% confidence interval will be calculated using this standard error.

Cluster adjusted kappa (EQ-5D-5L and HowRu domain levels) and ICC (EQ-VAS and EQ-5D index scores) will be reported for each time point. However, QALYs will be presented over time, three months, similar to how it will be calculated in the PEACH study.

Sample size calculation

We need a sample size of 160 residents assuming a kappa of 0.145 and a confidence level width of 0.153 derived from a previous study (16), given that 50% of residents will have any problems.

Secondary analyses

The effect of age, sex and length of stay in care home (for residents), length time working in care of older people/care homes and role/rank (for staff) at baseline on the difference between staff and proxy EQ-5D-5L-S scores will be analysed using a multilevel mixed effect regression model.

For peer review only - http://bmjopen.bmj.com/site/about/guidelines.xhtml

To investigate the reliability of using HowRu as a quality of life measure in the care home population compared with EQ-5D-5L, we will assess agreement between these indices using weighted kappa statistics. This will involve testing the level of agreement between domains with similar construct on both scales (31) as shown in table 1.

Table 1 - Planned pair-wise alignment of HowRU and EQ-5D-5L domains for agreement analysis using kappa

HowRu domains	EQ-5D-5L domains
Pain or discomfort	Pain/discomfort
Feeling low or worried	Anxiety/depression
Limited in what you can do	Mobility<br>Self-care<br>Usual activities
Dependent on others	Mobility<br>Self-care<br>Usual activities

Patient and Public Involvement

The APRICOT and PEACH studies were developed and designed in discussion with both Care Home Sector and Patient and Public Involvement (PPI) representatives. The initial research proposal and protocol was presented, prior to submission for funding, to the Dementia and Frail Older Person's PPI group hosted in the Division of Rehabilitation and Ageing, University of Nottingham. Amendments were made to the proposal and protocol in light of their feedback. The PEACH study team includes one PPI and two care home sector representatives who are present at all study management meetings, with oversight for the APRICOT sub-study. We keep all participating care homes working with PEACH updated through quarterly newsletters which will include dissemination of our findings in lay terms as these become available.

Strengths and Limitations of this study

- To our knowledge, this is the first study to investigate the reliability of staff proxy using the EQ-5D-5L and HowRu, and the reliability of HowRu as a quality of life measure in a UK care home setting.
- The scales will be administered to residents and staff on the same day, thus reducing any variations that will affect the level of agreement.
- Analysing the characteristics of staff respondents will help generate insights into how proxies might better be selected or prepared to maximise the validity of proxy responses.
- The use of multiple time points will help increase the sample size and account for the influence of staff turnover on the quality of proxy-response.
- Working with residents with mental capacity will optimise their responses as a gold standard against which proxies can be compared. The way in which staff interpret quality of life in this more communicative and able group may, however, be different to the way in which the interpret quality of life in advanced dementia.

For peer review only - http://bmjopen.bmj.com/site/about/guidelines.xhtml

Ethics and dissemination

This study is part of preparatory work for the larger PEACH study. PEACH was reviewed by both the UK Health Research Authority and the University of Nottingham Research Ethics Committee and determined by both to be a service development and evaluation project. We will obtain informed consent from residents who have mental capacity and use a consultee when residents lack mental capacity. The PEACH study protocol has been reviewed as part of good governance by the Nottinghamshire Healthcare Foundation Trust. We aim to publish this study in a peer-reviewed journal with international readership and disseminate it further using relevant national stakeholder networks and specialist societies.

Funding

This work has been conducted as part of the Proactive Healthcare of Older People in Care Homes (PEACH) study, supported by the Dunhill Medical Trust, award number FOP1/0115.

Contributors

All authors meet the ICJME criteria for authorship. AU, SL, KHS, AL, GH, JJ, HG, TD, JRFG and ALG conceived the study at a PEACH study management meeting and specified the aims and objectives. AU, SL and ALG produced the initial draft of the protocol, with KHS, AL, GH, JJ, HG, TD and JRFG contributing to subsequent redrafts. AU and SL led on aspects of statistical design. JJ and HG provided specialist health economics input. AU, SL, KHS, AL, GH, JJ, HG, TD, JRFG and ALG all reviewed the final manuscript and approved it prior to submission.

Competing interests

None declared.

Acknowledgements

The authors would like to acknowledge the broader PEACH study team: Mr Zimran Alam, Ms Anita Astle, Professor Tony Avery, Dr Jaydip Banerjee, Professor Clive Bowman, Dr Neil H Chadborn, Mr Michael Crossley, Dr Reena Devi, Professor Claire Goodman, Professor Pip Logan, Professor Finbarr Martin, Professor Julienne Meyer, Dr Dominick Shaw, Professor David Stott and Dr Maria Zubair.

References

1. Aspden T, Bradshaw SA, Playford ED, Riazi A. Quality-of-life measures for use within care homes: a systematic review of their measurement properties. Age and Ageing. 2014; 43(5):596-603.
2. Sanford AM, Orrell M, Tolson D, Abbatecola AM, Arai H, Bauer JM, et al. An International Definition for “Nursing Home”. Journal of the American Medical Directors Association. 2015;16(3):181-184.
3. Laing & Buisson. Care of the Elderly People Market Survey. London: Laing and Buisson 2010; 2009.
4. Gordon AL, Franklin M, Bradshaw L, Logan P, Elliott R, Gladman JRF. Health status of UK care home residents: a cohort study. Age and Ageing. 2014;43(1):97-103.

For peer review only - http://bmjopen.bmj.com/site/about/guidelines.xhtml

5. Kinley J, Hockley J, Stone L, Dewey M, Hansford P, Stewart R, et al. The provision of care for residents dying in UK nursing care homes. Age and Ageing. 2014;43(3):375-379.
6. Kane RL, Kane RA, Bershadsky B, Degenholtz H, Kling K, Totten A, et al. Proxy sources for information on nursing home residents' quality of life. The journals of gerontology Series B, Psychological sciences and social sciences. 2005;60(6):S318-s325.
7. Devlin N, Shah K, Feng Y, Mulhern B, van Hout B. Valuing health-related quality of Life: An EQ-5D-5L Value Set for England. Health Economics. 2017; 1-16.
8. Whitehead SJ, Ali S. Health outcomes in economic evaluation: the QALY and utilities. Br Med Bull. 2010;96(1):5–21.
9. McCabe C, Claxton K, Culyer AJ. The NICE Cost-EffectivenessThreshold. Pharmacoeconomics. 2008;26(9):733-744.
10. Janssen MF, Simon Pickard A, Golicki D, Gudex C, Niewada M et al. Measurement properties of the EQ-5D-5L compared to the EQ-5D-3L across eight patient groups: a multi-country study. Qual Life Res. 2013; 22(7): 1717-27
11. Borowiak E, Kostka T. Predictors of quality of life in older people living at home and in institutions. Aging Clinical and Experimental Research. 2004;16(3):212-220.
12. Devlin NJ, Brooks R. EQ-5D and the EuroQol Group: Past, Present and Future. Appl Health Econ Health Policy. 2017;15(2):127–37.
13. Parkin D, Devlin N. Is there a case for using visual analogue scale valuations in cost-utility analysis? Health Econ [Internet]. 2006 Jul 1 [cited 2018 Apr 25];15(7):653–64. Available from: http://doi.wiley.com/10.1002/hec.1086
14. Feng Y, Parkin D, Devlin NJ. Assessing the performance of the EQ-VAS in the NHS PROMs programme. Qual Life Res. 2014; 23(3):977–89.
15. Hounsome N, Orrell M, Edwards RT. EQ-5D as a Quality of Life Measure in People with Dementia and Their Carers: Evidence and Key Issues. Value in Health. 2011;14(2):390-399.
16. Devine A, Taylor SJC, Spencer A, Diaz-Ordaz K, Eldridge S, Underwood M. The agreement between proxy and self-completed EQ-5D for care home residents was better for index scores than individual domains. Journal of Clinical Epidemiology. 2014;67(9):1035-1043.
17. Bulamu NB, Kaambwa B, Ratcliffe J. A systematic review of instruments for measuring outcomes in economic evaluation within aged care. Health and Quality of Life Outcomes. 2015;13(1).
18. van Leeuwen KM, Bosmans JE, Jansen AP, Hoogendijk EO, van Tulder MW, van der Horst HE, et al. Comparing Measurement Properties of the EQ-5D-3L, ICECAP-O, and ASCOT in Frail Older Adults. Value in health : the Journal of the International Society for Pharmacoeconomics and Outcomes Research. 2015;18(1):35-43.
19. Benson T, Williams DH, Potts HWW. Performance of EQ-5D, howRu and Oxford hip & knee scores in assessing the outcome of hip and knee replacements. BMC Health Services Research. 2016;16(1).
20. Benson T, Potts HWW, Whatling JM, Patterson D. Comparison of howRU and EQ-5D measures of health-related quality of life in an outpatient clinic. Inform Prim Care. 2013;21(1):12-17.
21. Hendriks SH, Rutgers J, van Dijk PR, Groenier KH, Bilo HJG, Kleefstra N, et al. Validation of the howRu and howRwe questionnaires at the individual patient level. BMC Health Services Research. 2015;15(1).
22. Welsh TJ, Gordon AL, Gladman JR. Comprehensive geriatric assessment - a guide for the non-specialist. *Int J Clin Pract*. 2014. 68(3): 290–293
23. Dobson C. Conducting research with people not having the capacity to consent to their participation. A practical guide for researchers [Internet]. Leicester, UK: The

For peer review only

For peer review only - http://bmjopen.bmj.com/site/about/guidelines.xhtml

British Psychological Society; 2017 [cited 1 December 2017]. Available from: https://pdfs.semanticscholar.org/f97c/5a3d8605e24eaaf965b64e42c690d42a6182.pdf

24. Selai C, Pickard S, Johnston JA. The collection of EuroQol EQ-5D data via proxies. EQ Plenary Meeting in York: September, 2002

25. van Hout B, Janssen MF, Feng Y-S, Kohlmann T, Busschbach J, Golicki D, et al. Interim Scoring for the EQ-5D-5L: Mapping the EQ-5D-5L to EQ-5D-3L Value Sets. Value Heal.;15(5):708–15.

26. Vanbelle S, Albert A. A note on the linearly weighted kappa coefficient for ordinal scales. Statistical Methodology. 2009; 6(2):157-163.

27. Brenner H, Kliebsch U. Dependence of Weighted Kappa Coefficients on the Number of Categories. Epidemiology. 1996;7(2):199-202.

28. Cicchetti DV, Allison T. A New Procedure for Assessing Reliability of Scoring EEG Sleep Recordings. American Journal of EEG Technology. 1971;11(3):101-110.

29. Landis JR, Koch GG. The measurement of observer agreement for categorical data. Biometrics. 1977;33(1):159.

30. Harwell MR, Rubinstein EN, Hayes WS, Olds CC. Summarizing Monte Carlo Results in Methodological Research: The One- and Two-Factor Fixed Effects ANOVA Cases. J Educ Stat. 1992 Dec 23;17(4):315–39.

31. Kerry SM, Bland JM. Unequal cluster sizes for trials in English and Welsh general practice: implications for sample size calculations. Statistics in Medicine. 2001;20(3):377-

For peer review only

For peer review only - http://bmjopen.bmj.com/site/about/guidelines.xhtml

For peer review only

For peer review only - http://bmjopen.bmj.com/site/about/guidelines.xhtml
